# Do People Take Stimulus Correlations into Account in Visual Search?

**DOI:** 10.1371/journal.pone.0149402

**Published:** 2016-03-10

**Authors:** Manisha Bhardwaj, Ronald van den Berg, Wei Ji Ma, Krešimir Josić

**Affiliations:** 1 Department of Mathematics, University of Houston, Houston, Texas, United States of America; 2 Department of Neuroscience, Baylor College of Medicine, Houston, Texas, United States of America; 3 Department of Psychology, Uppsala University, Uppsala, Sweden; 4 Center for Neural Science and Department of Psychology, New York University, New York, New York, United States of America; 5 Department of Biology and Biochemistry, University of Houston, Houston, Texas, United States of America; University of Bath, UNITED KINGDOM

## Abstract

In laboratory visual search experiments, distractors are often statistically independent of each other. However, stimuli in more naturalistic settings are often correlated and rarely independent. Here, we examine whether human observers take stimulus correlations into account in orientation target detection. We find that they do, although probably not optimally. In particular, it seems that low distractor correlations are overestimated. Our results might contribute to bridging the gap between artificial and natural visual search tasks.

## Introduction

Visual target detection in displays consisting of multiple simple stimuli is a mainstay in visual science. Within this group of tasks, two classes can be distinguished: ones in which the distractors are identical to each other (homogeneous), and ones in which they are not (heterogeneous) [1]. Models have focused on homogeneous-distractor tasks, in which the value of the distractors is fixed across trials [2–9]. For example, an observer might be detecting a vertically oriented target among distractors that are always tilted 5° clockwise, or a signal among *N* image patches that otherwise consist of only pixel noise. In such conditions, human performance is well described by either a model in which the observer uses a maximum-of-outputs rule [9, 10] or a Bayesian maximum-a-posteriori rule [4, 10]. In another type of homogeneous-distractor task, the distractors are identical to each other but their value varies across trials [11]. A limitation of studies using homogeneous distractor sets is that stimuli outside the laboratory are often heterogeneous. For example, when detecting an animal hidden in the bushes, a friend in a crowd, keys in a cluttered drawer, or a tumor on a CT scan, distractors typically vary in their features both across space and across time. Modeling work on heterogeneous search—which has not been as extensive as modeling of homogeneous search—has found that a Bayesian-observer model provides a good description of human search for a fixed target among distractors that are drawn independently from either a uniform [12, 13] or a normal distribution [11, 14] (although perhaps less so when the distribution is more complex [15]).

The assumption of independent distractor is probably not correct in more naturalistic settings, where distracting elements will often have structure amongst themselves and therefore be correlated. Here, we ask whether human observers take stimulus correlations into account when detecting a target among distractors, and in particular whether they may assume correlations where none exist, as has been observed in other contexts [16–19]. We find that human observers take correlations into account, but indeed overestimate low correlations.

## Experimental Methods

### Task

**Fig 1:**
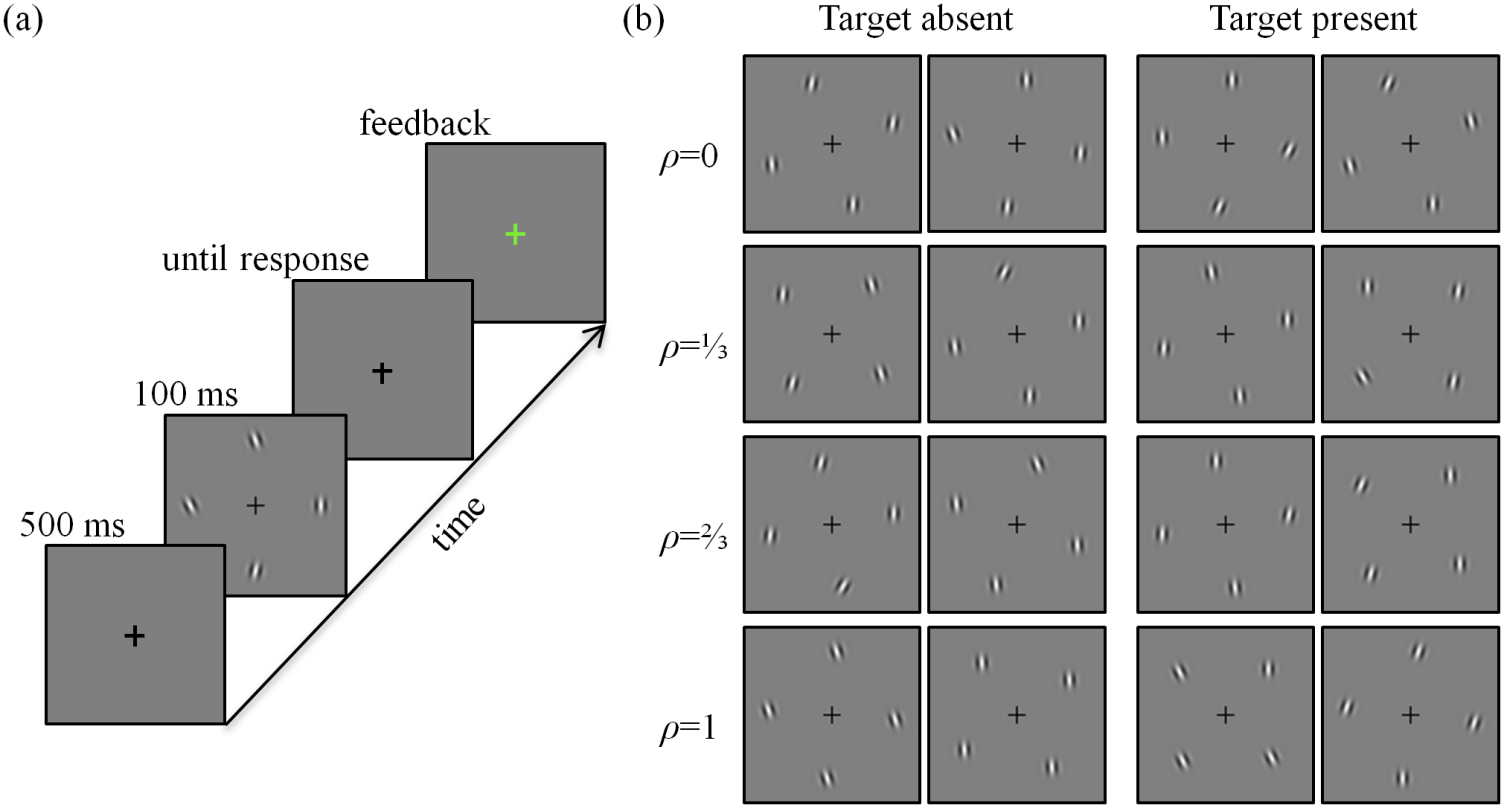
Experimental procedure and sample displays. (a) Time course of a trial, (b) Sample displays for each of the correlation coefficients used. In a given experimental session, only one value of *ρ* was used.

We conducted a target detection experiment in which observers were presented with four oriented Gabor patches (Fig 1). The search target was a vertically oriented Gabor patch and was present with probability 0.5 at a randomly chosen location. The task of the observers was to report on each trial whether the target was present. We refer to orientations of patches that were not the target as “distractors”. Distractor orientations were drawn from a multivariate normal distribution. The marginal distribution of each distractor had a mean of 0° (vertical) and a standard deviation of 15°. The amount of structure within a display was controlled by the correlation coefficient, *ρ*, between distractor orientations. We used uniform correlations, which mean that *ρ* was the same for all distractor pairs (Fig 1b). In a given experimental session, *ρ* took one of four values: 0 (independent distractors), ⅓, ⅔, or 1 (identical distractors).

### Subjects

Eleven subjects (6 male, 5 female) participated in the experiment. All subjects had normal or corrected-to-normal acuity and gave written informed consent. The study was approved by the Institutional Review Board of the Baylor College of Medicine, Houston, Texas.

### Apparatus and stimuli

Stimuli were presented on a 21″ LCD monitor with a refresh rate of 60 Hz. Subjects viewed the displays from a distance of approximately 60 cm. The background luminance was 33.1 cd/m^2^. A set of 4 stimuli was shown on each trial. On target-present trials, the stimulus set consisted of 1 target and 3 distractors while on target-absent trials, it consisted of 4 distractors. A target was present on exactly half the trials. Each stimulus was a Gabor patch with a spatial frequency of approximately 2.67 cycles/deg, a standard deviation of 0.26 deg, and a peak luminance of 136 cd/m^2^ (which corresponds to a Michelson contrast of 0.61). Stimuli were placed on an invisible circle centered at the fixation cross, with a radius of 3.2 degrees of visual angle. On each trial, the first stimulus was placed at a random position along the circle, and the other stimuli were placed so that the angular distance between two adjacent stimuli was always 45°. On target-present trials, each location was equally likely to contain the target. The standard deviation of the distractor distribution, *σ*_s_, was fixed at 15° while the correlation coefficient, *ρ*, was varied across different experimental sessions.

### Procedure

Each subject participated in four sessions. Each session lasted about 50 minutes and was run on a different day or on the same day with an interval of at least an hour between consecutive sessions. No more than two sessions were run on a single day for a subject. Within each session the correlation coefficient *ρ* was fixed at one of the values 0, ⅓, ⅔, or 1. The order of the sessions was randomized across subjects. Each session consisted of one training block of 50 trials and 6 testing blocks of 150 trials each. Each training trial began with the display of a fixation cross at the center of the screen (500 ms), followed by the stimulus display containing 4 stimuli (100 ms). After the stimuli were presented, only the fixation cross was displayed until the subject responded (Fig 1a). Subjects reported through a key press whether the target was present or absent. After each response, feedback was provided by coloring the fixation cross green (correct) or red (incorrect) for 750 ms. During training, this was followed by a second presentation of the stimuli for 2 s, with a blue circle identifying the target stimulus if one was present. Testing trials were identical to training trials, except that feedback was provided only by changing the color of the fixation cross; the stimuli were not redisplayed. A subject’s performance was revealed after the completion of each block of 150 trials, along with the scores of the other subjects who had completed the same session. Each subject completed a total of 3600 test trials. At the beginning of the first session, we explained the trial procedure while demonstrating one training trial step by step. After that, the subject completed 9 more practice trials in the presence of the experimenter. At the end of the first session, we told the subject that in the next session, the type of display would be slightly different from what they had experienced in the first session. We never told subjects explicitly about correlations.

## Experimental Results

**Fig 2:**
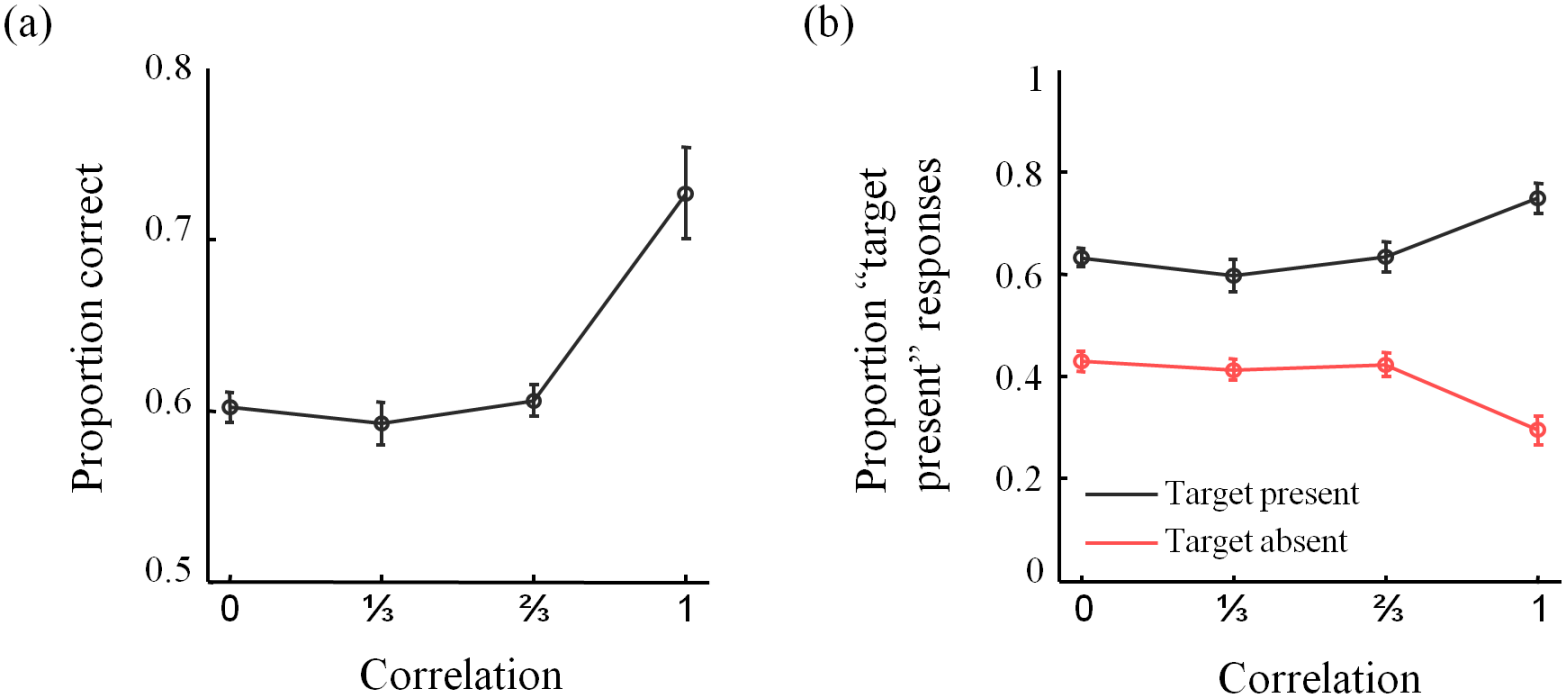
Psychometric curves 1. (a) Proportion correct responses and (b) hit and false alarm rates as a function of distractor correlation. Throughout the paper, error bars indicate one standard error of the mean (s.e.m).

Distractor correlation had a significant effect on the proportion of correct responses (repeated-measures ANOVA: *F*(3,40) = 15.75, *p*<0.0001; Fig 2a). This effect is still present when the hit and false alarm rates are analyzed separately (hit rate: *F*(3,40) = 5.57, *p* = 0.0027; false-alarm rate: *F*(3,40) = 8.14, *p* = 0.0002; Fig 2b) and seems to be mostly driven by the performance increase in the *ρ* = 1 condition. To visualize the subject data and model fits, we computed two summary statistics, separately for each *ρ*-condition: the proportion of target present responses as function of both the standard deviation of the distractor set and as function of the minimum difference between the orientation of the target and any distractor.

**Fig 3:**
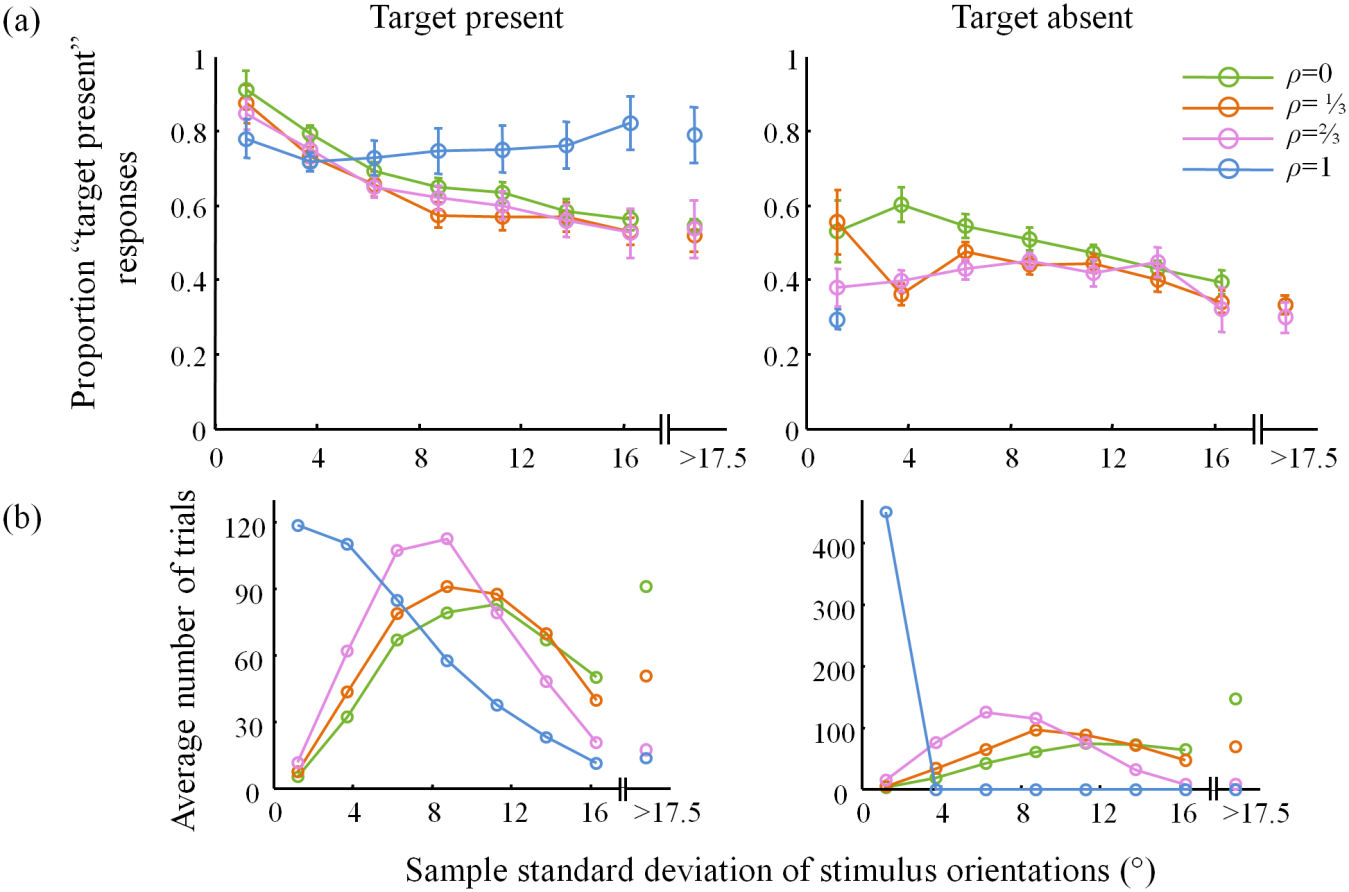
Psychometric curves 2. (a) Proportion “target present” responses and (b) number of trials as a function of standard deviation of the distractor set coefficient, averaged across subjects. Bin size was 2.5°, except that all trials with sample standard deviation greater than 17.5° are collected in the last bin. The plots in (b) are entirely determined by the stimuli, not by the subject responses; they serve to emphasize that the points in the plots in (a) were computed on widely differing numbers of trials.

On average, the proportion of “target present” responses decreases as a function of the standard deviation of the distractor set, except in the *ρ* = 1 condition (Fig 3a). Note that the number of trials per bin differs both across bins and across correlation conditions (Fig 3b). In particular, in the target-absent trials, the standard deviation of a distractor set in the homogeneous condition (*ρ* = 1) is always zero.

**Fig 4:**
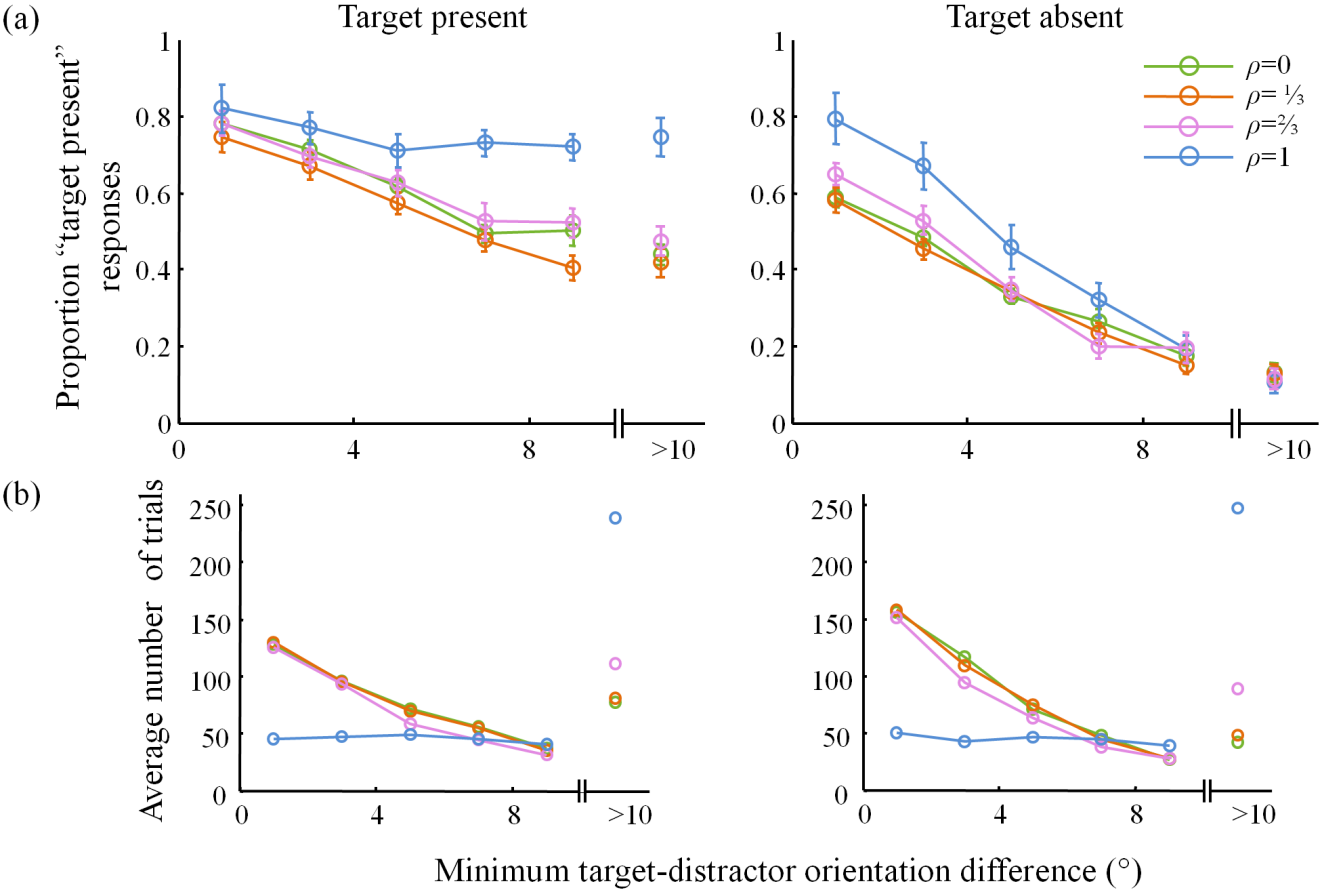
Psychometric curves 3. (a) Proportion “target present” responses and (b) number of trials as a function of minimum target-distractor orientation difference for target present (left) and absent (right) trials. Bin size was 2°, except that all trials with minimum target-distractor orientation difference greater than 10° are collected in the last bin. The plots in (b) are entirely determined by the stimuli, not by the subject responses; they help to reconcile the plots in (a) with Fig 2b.

Similarly, the proportion of “target present” responses generally decreases with the minimum angle between the distractors and the (vertical) target orientation (Fig 4a). The differences in the numbers of trials per bin (Fig 4b) produce a paradox: for example, in the target-absent condition (Fig 4a, right), the entire *ρ* = 1 curve lies above the *ρ* = 0 curve, even though subjects respond “target present” overall less in the *ρ* = 1 condition (Fig 2b). This is an instance of Simpson’s paradox [20]; it is resolved by realizing that the trials in the *ρ* = 0 are heavily weighted towards bins corresponding to smaller values (Fig 4b, right).

## Models

To determine whether and how subjects took correlations into account in this visual search task, we fitted the optimal-observer model and several alternative models to the data. Here, we first describe the generative model—which specifies how observations are statistically related to the stimuli—in its most general form. We then derive the optimal decision rule. Finally, we give an overview of the models that we fitted to the data. All models are variations of the optimal-observer model.

### Specification of the generative model

The first step of Bayesian modeling is to define the task-relevant random variables and their dependencies, collectively called the generative model. Although the number of stimuli, *N*, was always 4 in our experiment, we present our model for general *N*. We denote target presence by a binary variable *T*, with *T* = 0 denoting "target absent" and *T* = 1 denoting "target present". The probability of target presence, *p*(*T* = 1), is equal to 0.5. When *T* = 1, a target location is chosen with uniform probability. The target orientation is always vertical, which we define as 0°. We denote the vector of stimulus orientations by **s** = (*s*_1_,…,*s*_*N*_).

On a target-absent trial, **s** is drawn from a *N*-dimensional multivariate normal distribution with mean (0,…,0) and covariance **Σ**_s_, which for *N* = 4 is

$$\Sigma_{\text{s}}=\sigma_{\text{s}}^{2}\left(\begin{array}{cccc} 1 & \rho & \rho & \rho \\ \rho & 1 & \rho & \rho \\ \rho & \rho & 1 & \rho \\ \rho & \rho & \rho & 1 \end{array}\right).$$

Here, the correlation coefficient *ρ* is between 0 and 1. When *ρ* = 0, the orientations of all distractors are chosen independently (maximal heterogeneity); when *ρ* = 1, they are identical (homogeneous). This design interpolates between the homogeneous and heterogeneous conditions in an earlier study [11].

On a target-present trial with target at location *j*, the orientations of the *N*-1 distractors, **s**_\j_ = (*s*_1_,…,*s*_j-1_,*s*_j+1_,…,*s*_*N*_), are drawn from a (*N*-1)-dimensional multivariate normal distribution with mean, **0**_N-1_ = (0,…,0) and covariance, **Σ**_s\j_. The notation \*j* refers to the set of distractors when the target is present at location *j*. The (*N*-1)×(*N*-1) covariance matrix, **Σ**_s\j_, is obtained by removing the *j*^th^ row and the *j*^th^ column of **Σ**_s_, and we write

p(s\j|T=1) = N (s\j;0N−1,Σs\j)

We denote the observer's vector of stimulus measurements by **x** = (*x*_1_,…, *x*_N_). We assume that the stimulus measurements are corrupted by zero-mean Gaussian noise, so that, for the *i*^th^ location, we have

p(xi|si) = N (xi;si,σi2).

We further assume that measurement noise is independent between locations.

### Optimal decision rule

Optimal observers infer whether a target is present or not from the stimulus measurements, **x**, by using their knowledge of the generative model. Specifically, an optimal observer computes *p*(*T* = 1|**x**) and *p*(*T* = 0|**x**) and reports which possibility is more probable. This is equivalent to computing the log posterior ratio,

$$d=\text{log}\frac{p\left(T=1|\text{x}\right)}{p\left(T=0|\text{x}\right)}=\text{log}\frac{p\left(\text{x}|T=1\right)}{p\left(\text{x}|T=0\right)}+\text{log}\frac{p\left(T=1\right)}{p\left(T=0\right)},$$ (1)

and reporting "target present" if *d*>0 and "target absent" otherwise. If the optimal observer assumes equal probabilities for *T* = 0 and *T* = 1, then we find that *d* is given by (see S1A Appendix)

$$d=\text{log}\left(\frac{1}{N}\sum_{j=1}^{N}\sqrt{\frac{J_{j}\left(1+\rho \sigma_{s}^{2}\sum_{i=1}^{N}{\tilde{J}}_{i}\right)}{{\tilde{J}}_{j}\left(1+\rho \sigma_{s}^{2}\sum_{i\neq j}^{N}{\tilde{J}}_{i}\right)}}\text{exp}\left(-\frac{1}{2}\left(\left(J_{j}-{\tilde{J}}_{j}+\alpha {\tilde{J}}_{j}^{2}\right)x_{j}^{2}+{\tilde{J}}_{j}\alpha x_{j}\sum_{i\neq j}^{N}{\tilde{J}}_{i}x_{i}-\left(\alpha_{\backslash j}-\alpha \right)\sum_{i,k\neq j}^{N}{\tilde{J}}_{i}{\tilde{J}}_{k}x_{i}x_{k}\right)\right)\right)$$

where

$$\begin{array}{l} J_{i}=\frac{1}{\sigma_{i}^{2}},\\ {\tilde{J}}_{i}=\frac{1}{\sigma_{i}^{2}+\sigma_{\text{s}}^{2}\left(1-\rho \right)},\\ \alpha =\frac{\rho \sigma_{\text{s}}^{2}}{1+\rho \sigma_{\text{s}}^{2}\sum_{i=1}^{N}{\tilde{J}}_{i}},\;\text{and}\\ \alpha_{\backslash j}=\frac{\rho \sigma_{\text{s}}^{2}}{1+\rho \sigma_{\text{s}}^{2}\sum_{i\neq j}^{N}{\tilde{J}}_{i}}. \end{array}$$

Thus, the decision variable maps the stimulus measurements, **x**, and the variances of the noise in each measurement, *σ*_1_^2^,…, *σ*_*N*_^2^, to a real number. The dependence of the decision variable on the measurements is complex and difficult to interpret in general. However, the cases *ρ* = 0 and *ρ* = 1 are intuitive and tractable. When *ρ* = 0, distractor orientations are chosen independently, and the decision variable is given by:

$$d=\text{log}\left(\frac{1}{N}\overset{N}{\underset{j=1}{\sum }}\sqrt{\frac{\sigma_{j}^{2}+\sigma_{s}^{2}}{\sigma_{j}^{2}}}\text{exp}\left(-\frac{\sigma_{s}^{2}x_{j}^{2}}{2\sigma_{j}^{2}\left(\sigma_{j}^{2}+\sigma_{s}^{2}\right)}\right)\right).$$

In this case, the optimal observer makes a decision based on a weighted average of all stimulus measurements [11]. The weights are determined by the uncertainty of each measurement. A measurement closer to 0 provides stronger evidence that a target is present. When *ρ* = 1, all distractors are identical and the decision variable is given by

$$d=\text{log}\left(\frac{1}{N}\overset{N}{\underset{j=1}{\sum }}\sqrt{1+J_{j}\alpha_{\backslash j}}\text{exp}\left(-\frac{1}{2}\left(\alpha {\left(\overset{N}{\underset{i=1}{\sum }}J_{i}x_{i}\right)}^{2}-\alpha_{\backslash j}{\left(\overset{N}{\underset{i\neq j}{\sum }}J_{i}x_{i}\right)}^{2}\right)\right)\right).$$

In this case, the optimal observer compares the squared weighted mean over all measurements to the squared weighted mean over all observations excluding a putative target [11]. Roughly speaking, if the *j*^th^ item is the target, the difference between these two quantities will be more negative than if the target is absent, so the exponential term is higher, contributing to the overall evidence for target presence.

So far, we have assumed that the observer knows that the frequencies of target-present and target-absent trials are equal, and incorporates this knowledge. We do not make this assumption in the models that we fit to data. Instead, we allow for the possibility that the observer behaves as if they believe that target-present trials occur with probability *p*(*T* = 1) = *p*_present_. As Eq 1 shows, this prior probability will appear in the expression for *d* as an additive term $\text{log}\frac{p_{\text{present}}}{1-p_{\text{present}}}$.

### Model overview

The models that we fitted to the data have two factors: the observer's assumption about the distractor correlations, and the presence/absence of variability in the precision of stimulus measurements. We considered four possibilities for the first factor and two for the second, giving a total of 8 models (Table 1).

**Table 1:** Summary of models. The models are organized according to two factors: the presence of variability in measurement precision (EP and VP), and the observer’s assumption about the correlation coefficients, **ρ**.

Precision	Model name	Observer’s assumption about ρ	Number of free parameters
EP	EP1	**ρ**_assumed_ **= ρ** = (0,⅓,⅔,1)	2 (*p*_present_ and *J*)
	EP2	**ρ**_assumed_ **=** (0,0,0,0)	2 (*p*_present_ and *J*)
	EP3	**ρ**_assumed_ **=** (*α*,*α*,*α*,*α*)	3 (*p*_present_, *J*, and *α*)
	EP4	**ρ**_assumed_ **=** (*α*,*β*,*γ*,*δ*)	6 (*p*_present_, *J*, *α*, *β*, *γ*, and *δ*)
VP	VP1	**ρ**_assumed_ **= ρ** = (0,⅓,⅔,1)	3 (*p*_present_, $\bar{J}$, and *τ*)
	VP2	**ρ**_assumed_ **=** (0,0,0,0)	3 (*p*_present_, $\bar{J}$, and *τ*)
	VP3	**ρ**_assumed_ **=** (*α*,*α*,*α*,*α*)	4 (*p*_present_, $\bar{J}$, *τ* and *α*)
	VP4	**ρ**_assumed_ **=** (*α*,*β*,*γ*,*δ*)	7 (*p*_present_, $\bar{J}$, *τ*, *α*, *β*, *γ*, and *δ*)

#### Observer’s assumption about *ρ*

An optimal observer has complete knowledge of the generative model, including the values of the correlations in all conditions, which we denote by a vector **ρ** = (0,⅓,⅔,1). There are other assumptions about **ρ** that an observer could be making, leading to suboptimal performance. We consider the following four possible assumptions:

- **ρ**_assumed_ = **ρ** = (0,⅓,⅔,1): the observer uses the correct values of distractor correlations (optimal).
- **ρ**_assumed_ = (0,0,0,0): the observer assumes that orientations are drawn independently of each other in all four conditions (which is optimal only in the first condition).
- **ρ**_assumed_ = (*α*,*α*,*α*,*α*): the observer assumes that the distractor correlation is the same in all four conditions. The assumed value for this correlation, *α*, is a free parameter fit to the data.
- **ρ**_assumed_ = (*α*,*β*,*γ*,*δ*): the observer assumes a different value for the distractor correlation across experimental conditions The assumed correlations, *α*, *β*, *γ*, and *δ* are free parameters.

#### Presence of variability in encoding precision

Recent studies have found evidence that the level of measurement noise can vary across trials and across locations within a trial [11, 13, 21–25]. Therefore, we considered two types of models:

- Equal-precision (EP) models, in which measurements have the same precision (inverse variance) across trials and stimuli. In this type of model measurement precision, *J*_*i*_ = *J* for all *i*.
- Variable-precision (VP) models, in which measurement precision is a random variable. In line with previous work [13, 23], we assumed that each element in the precision vector **J** = (*J*_1_,…,*J*_*N*_) follows a Gamma distribution with mean $\frac{\bar{J}}{\tau }$ and scale parameter *τ*. Note that $\bar{J}$, and *τ* are hyperparameters in the VP models. Each value in the vector is sampled independently across trials and stimuli.

## Model Comparison Results

Our main question is whether and how humans take into account stimulus correlations in visual search. A secondary question that we address is whether the current study supports the evidence for variability in encoding precision that we found in previous work on visual search [11, 13]. We first present results pertaining to the second question, because they turned out to be more clear-cut.

We used maximum-likelihood estimation to fit our 8 models to subject data (see S1B Appendix for details on the methods and S1 Table for parameter estimates). We compared models using the Akaike Information Criterion (AIC) and the Bayesian Information Criterion (BIC) (see S1C Appendix). A parameter recovery analysis (see S1B Appendix) showed that in our case, BIC recovers the correct model more reliably than AIC. We find that recovery is good, but that correlations tend to be biased away from the extreme values 0 and 1.

### Equal versus variable precision

**Fig 5:**
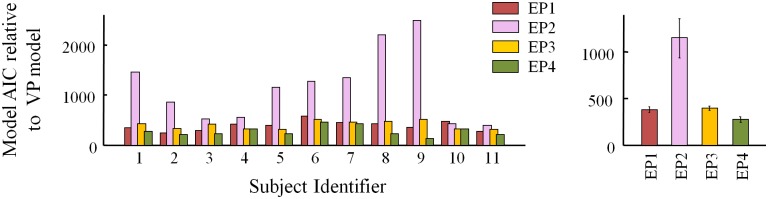
AIC model comparison for equal versus variable precision. Shown are AIC differences of EP models relative to VP models for each subject (left) and averaged over subjects (right). Higher AIC mean worse fits. BIC results are consistent (S1a Fig).

We compared the fit of each equal-precision model with its variable-precision counterpart. Fig 5 shows that regardless of the observer’s assumption about correlations, the variable-precision models better fit the data. This agrees with previous results [11, 13]. Therefore, we only consider the variable-precision models in further analyses.

### Do subjects take stimulus correlations into account?

**Fig 6:**
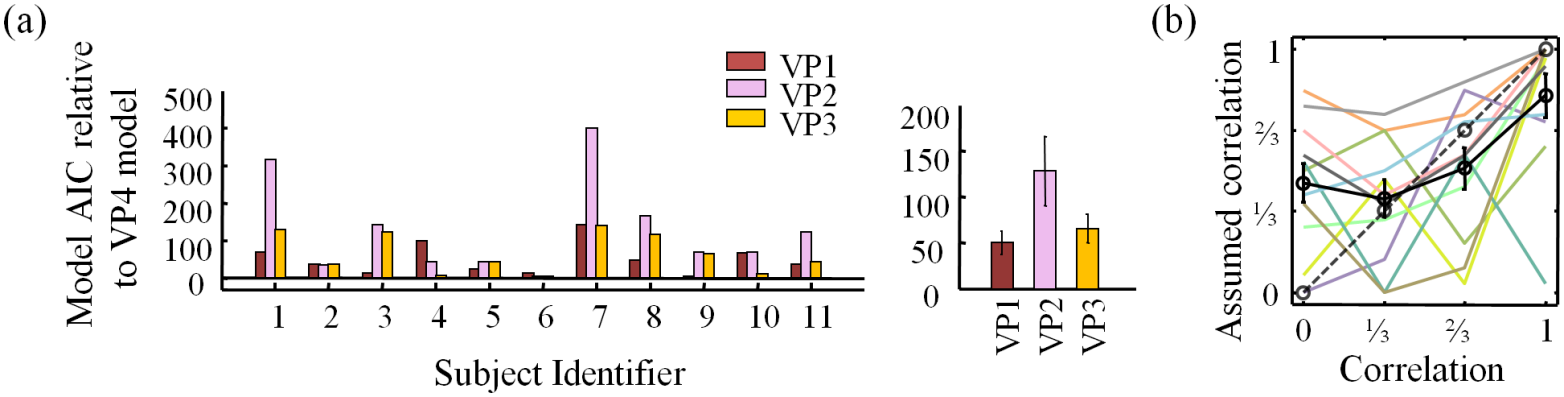
AIC model comparison of VP models for observer’s assumption about *ρ* and parameter estimates of VP4 model ρ_assumed_. (a) Shown are AIC differences of VP models relative to VP4 (most general) model for each subject (left) and averaged across subjects (right). (b) ML estimates of **ρ**_assumed_ from the VP4 model for each subject (colors) and averaged (black). BIC results are consistent (see S1b Fig).

We next examine whether subjects take into account correlations between distractor orientations when inferring the presence of a target. We found that suboptimal model VP4 provided the best fit to the data of each of the 11 subjects (Fig 6a). On average, the AIC value of the VP4 model was 50±13 lower than that of the optimal (VP1) model, which provides strong evidence against the hypothesis that human subjects take stimulus correlations into account in an optimal manner. Model VP4 also outperforms VP2 and VP3 (on average by 128±38 and 65±16, respectively), indicating that subjects do not assume zero or identical correlations across conditions. Hence, it seems that the subjects did take stimulus correlations into account in their decisions, but in a way that deviated substantially from the optimal strategy.

**Fig 7:**
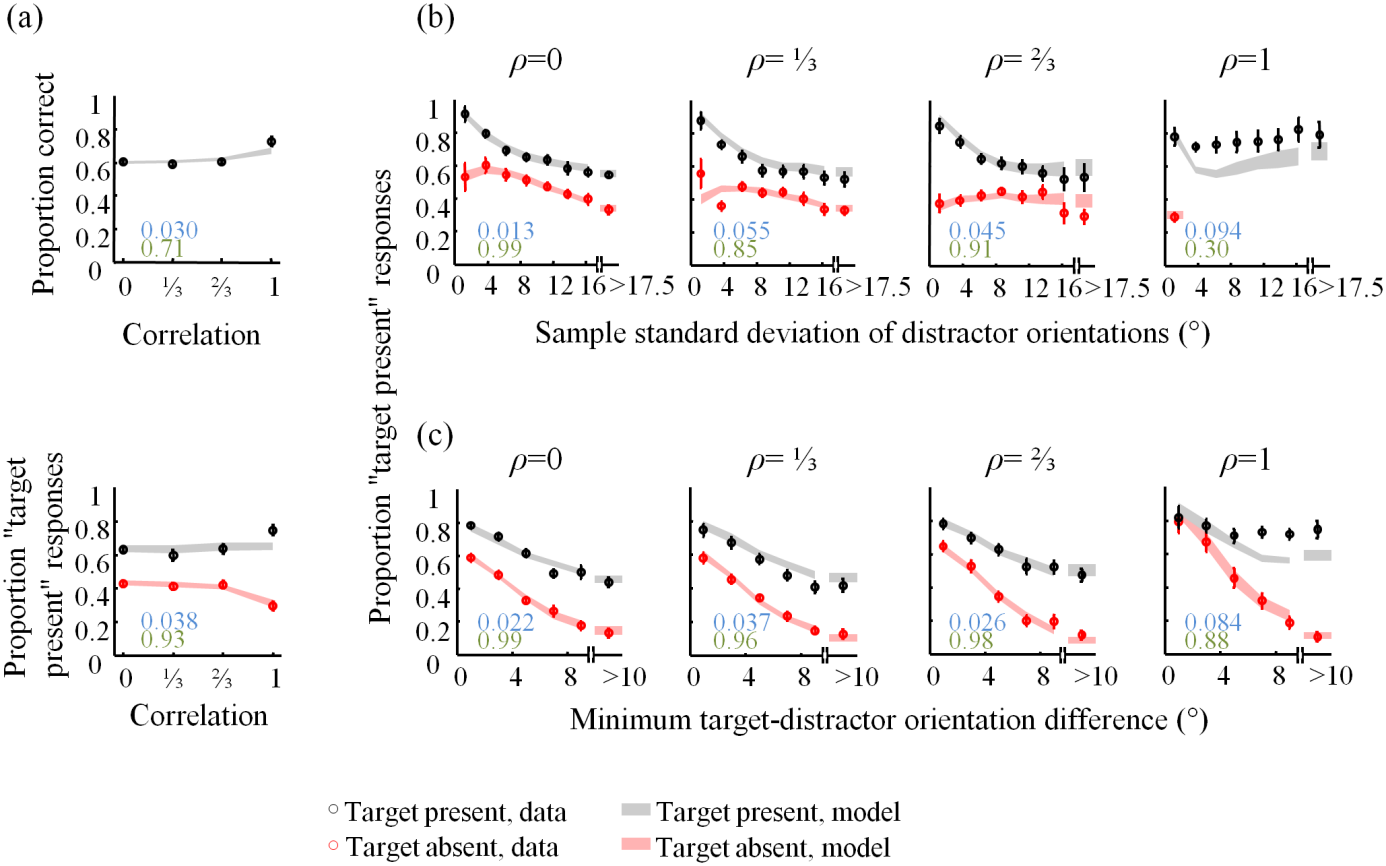
Fits of the VP4 model to the summary statistics. (a) Proportion correct (top), hit, and false-alarm rates (bottom) as a function of distractor correlation. Proportion “target present” responses as a function of (b) standard deviation of the distractor set, and (c) minimum target-distractor orientation difference, averaged across subjects, separately for target present (black) and target absent (red) trials. Numbers indicate root-mean square error (blue) and *R*^2^ statistics (green) between model and data.

The estimates of the observer's assumed values of the correlation coefficient in the VP4 model are shown in Fig 6b. While these estimates suggest that subjects overestimate low correlations and underestimate high ones, these estimates should be interpreted with caution, for the following reasons:

- both the uncertainty in the parameter estimates within a subject and the variability across subjects are large partly due to limited data;
- if a model does not fit well (as is the case in the *ρ* = 1 condition (Fig 7), its parameters are not meaningful;
- in synthetic data generated from the VP4 model, the correlation coefficient is also misestimated (see S1B Appendix), with a similar (but weaker) trend as in Fig 6b.

We can therefore conclude that human observers take correlations into account in this target detection task, however not optimally. Our models indicate that observers assume different correlations under different conditions, but we cannot say precisely what correlations they do assume.

### Model fits

**Fig 8:**
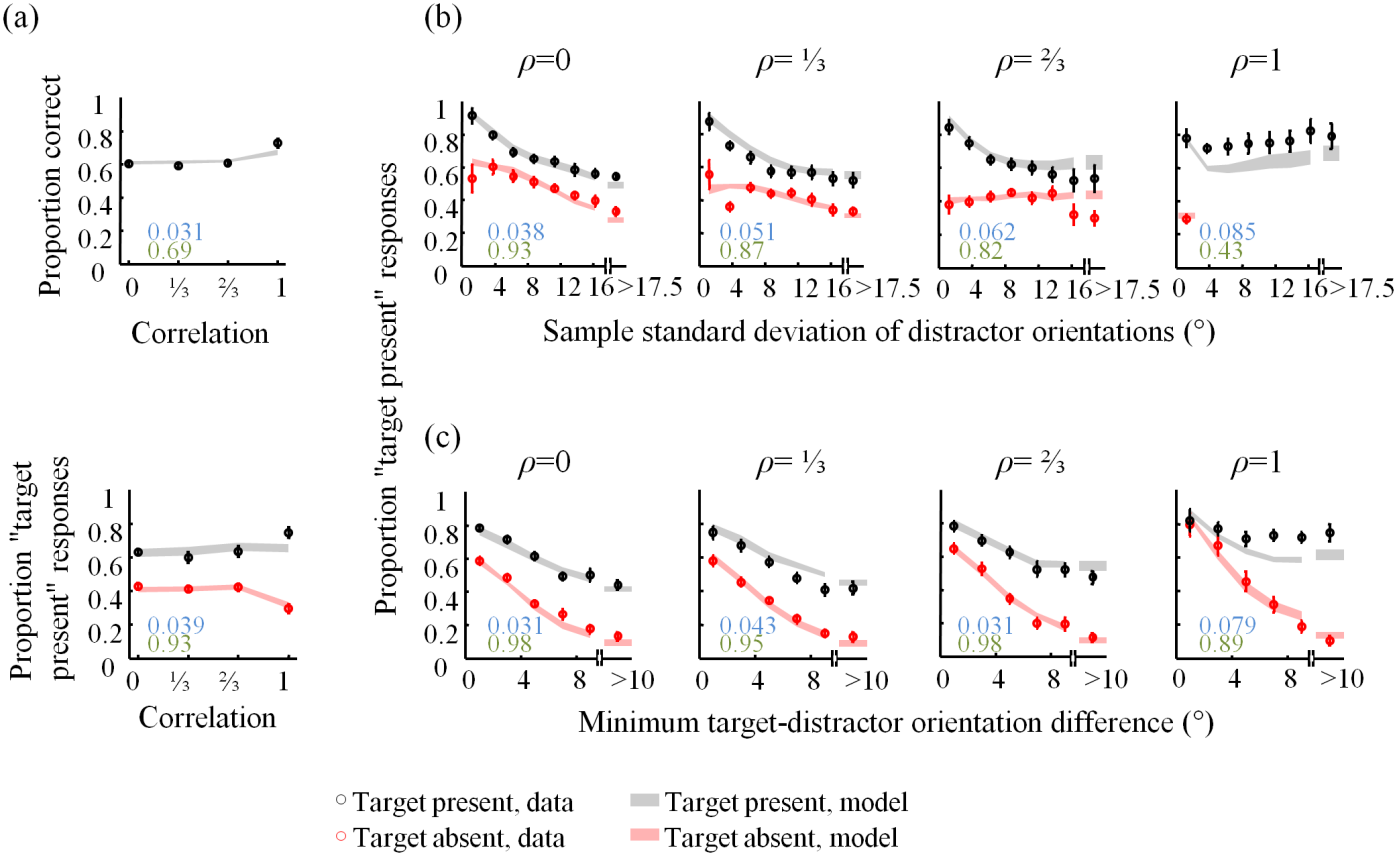
Fits of the VP1 model to the summary statistics. For caption, see Fig 7.

A model that wins in a model comparison does not necessarily fit the data well. To visualize the performance of our best model, VP4, we show how it fits the psychometric curves from Figs 2, 3 and 4 in Fig 7. For comparison, the fits of the optimal (VP1) model are shown in Fig 8. Although the VP4 model provides an overall better fit, it also deviates from the data in apparently systematic ways, especially in the homogeneous (*ρ* = 1) condition.

### Post-hoc models

Given how poorly the models fit in the *ρ* = 1 condition, we examined a post-hoc model in which mean precision,$\bar{J}$, depends on the correlation condition; we call this the VP5 model. Such a dependency might be justified if the items are not encoded independently, but as a configuration [26]. Alternatively, differences in $\bar{J}$ might reflect different degrees of suboptimality in an earlier stage of inference [27]. In spite of these justifications, the VP5 model is ad hoc.

**Fig 9:**
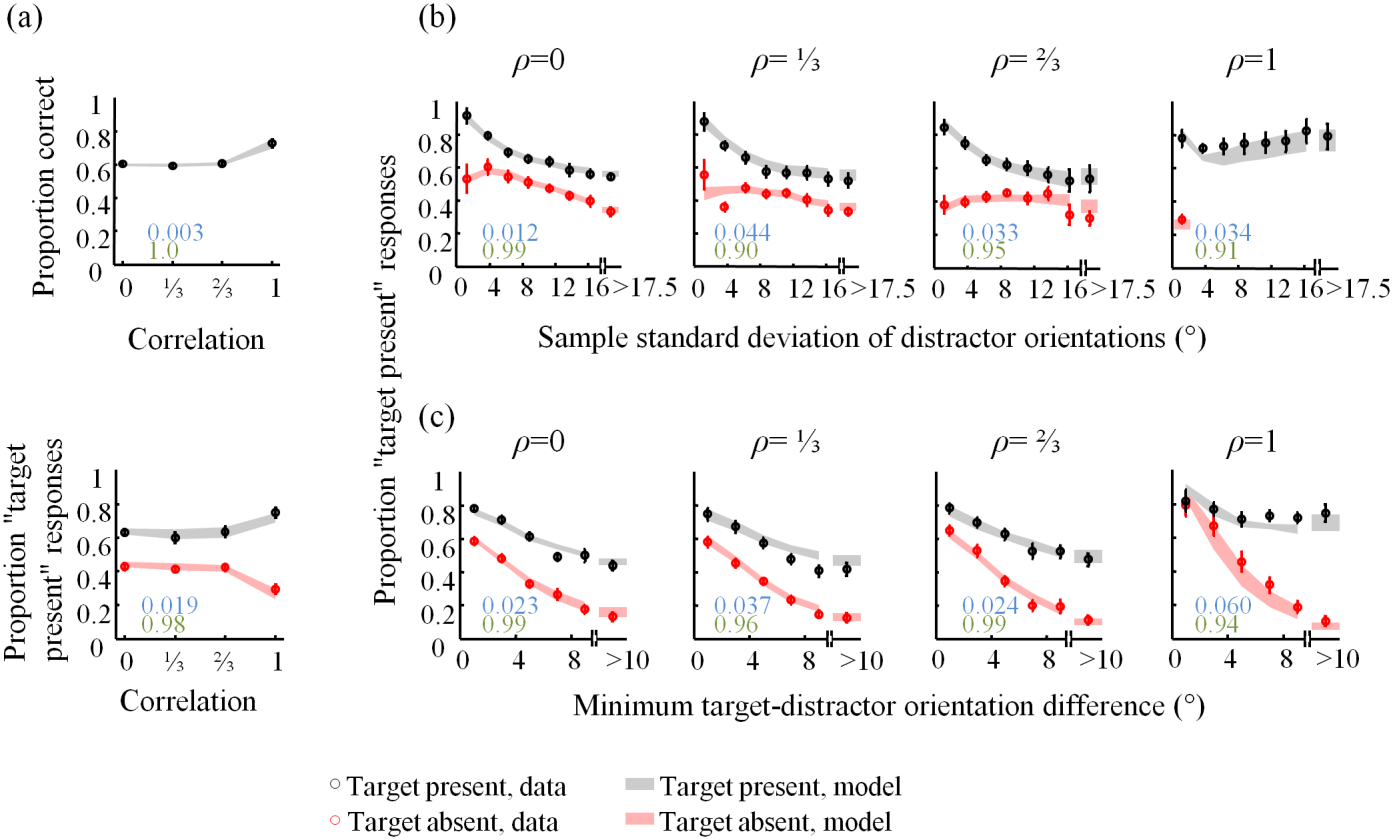
Fits of the VP5 model to the summary statistics. For caption, see Fig 7.

**Fig 10:**
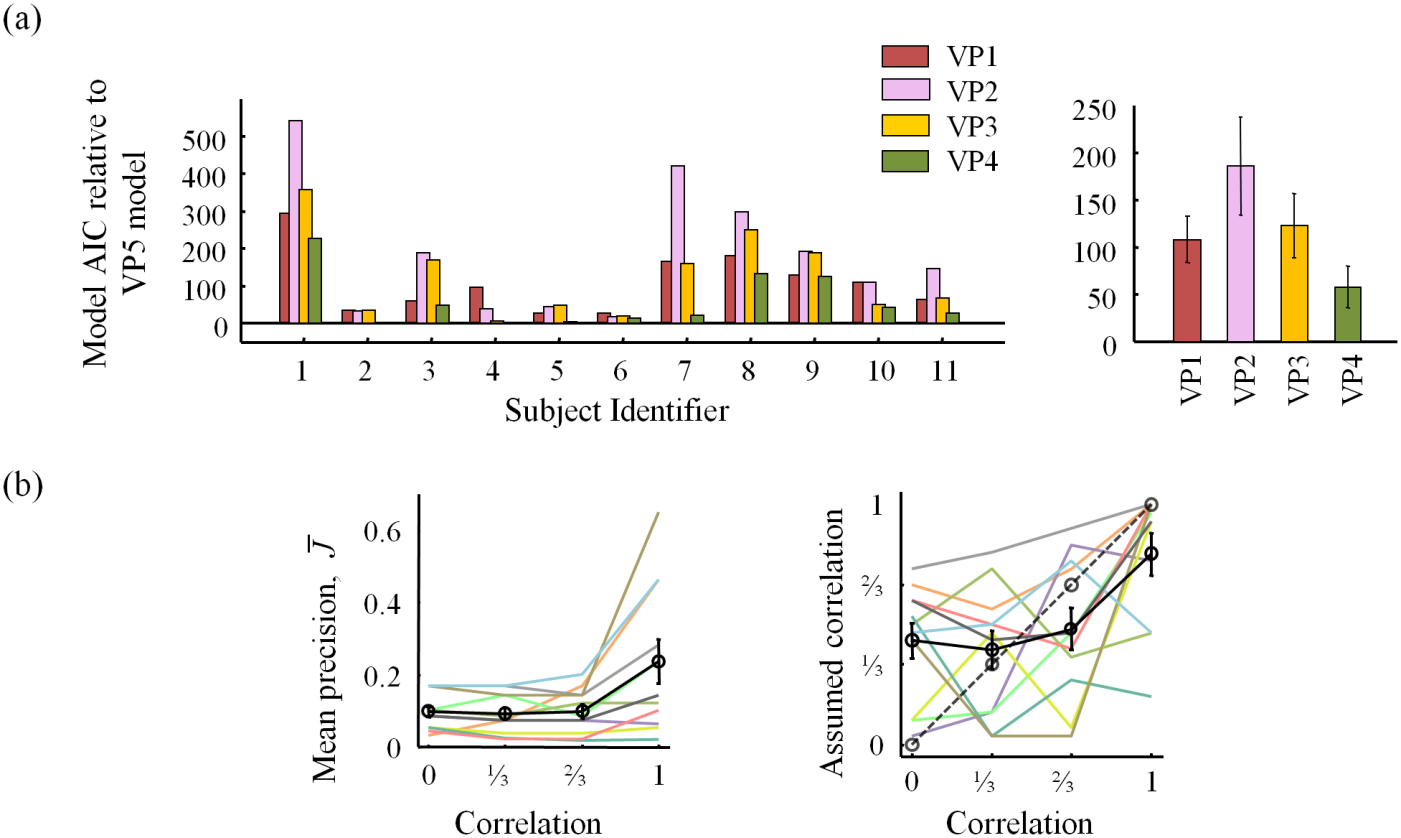
AIC model comparison of VP models relative to VP5 model and parameter estimates of VP5 model. (a) Shown are AIC differences of VP models relative to VP5 model for each subject (left) and averaged across subjects (right). BIC results are consistent (S1c Fig). (b) ML estimates of $\bar{J}$ and **ρ**_assumed_ from the VP5 model for each subject (colors) and averaged with standard error mean across subjects (black).

The VP5 model provides substantially better fits to summary statistics (Fig 9), particularly in the *ρ* = 1 condition. The VP5 model outperforms all other VP models in AIC: VP1 by 108±25, VP2 by 185±52, VP3 by 123±34, and VP4 by 58±22 (Fig 10a). Parameter estimates are shown in Fig 10b. Mean precision, $\bar{J}$, is estimated substantially higher in the *ρ* = 1 condition than in the other conditions, suggesting that homogeneous displays are encoded in a fundamentally different (more efficient) way than heterogeneous ones. Furthermore, **ρ**_assumed_ follows a similar relationship as in the VP4 model (Fig 6b). Hence, our conclusion regarding how subjects take correlations into account in this task does not strongly depend on the model that we fit. More experiments, potentially with different values of **ρ**, larger set sizes, and more extensive training could shed more light on how exactly people misestimate stimulus correlations in visual search.

## Discussion

The natural world is full of correlations between stimuli. Therefore, to understand how decisions are made in natural environments, it is necessary to go beyond independent stimuli typically used in psychophysics and study whether and how observers take into account stimulus correlations. There has been recent interest in this question. In contour integration, humans seem to be taking into account natural co-occurrence statistics of line elements [28]. In change detection, people incorporate knowledge about the large-scale statistical structure of a scene [26, 29]. It has been proposed that overestimation of correlations can explain set size effects [30][31].

Here, we tested the effect of introducing a nontrivial statistical structure in a visual search task by asking subjects to detect a vertical target among correlated distractors. Varying the correlation coefficient of the distractors allowed us to compare several models of human decision-making, all variants of the optimal-observer model. Within this set of models, we were able to rule out that the observer used the correct values of the correlations in the decision process. We were also able to rule out two suboptimal-observer assumptions about the correlations: that stimuli are uncorrelated, or that the correlations are constant. We found that the best model was the most flexible one, in which the assumed values of the correlations could differ between all correlation conditions. A similar conclusion has been reached in a study of human subjects in a reaching task in a three-dimensional virtual reality environment [32]. In that study, a strong correlation was induced between two dimensions of a randomly displaced target, which an optimal observer would learn to take into account. Human subjects did take these correlations into account, but not perfectly and to a degree that varied considerably between subjects. For later work, it should be kept in mind that observers might not have properly learned the joint distractor distribution in our experiment; this could be improved through explicit instructions, more training trials, or using more than four stimuli (so that observers have a larger sample to estimate the correlation).

Our best-fitting models (VP4 and VP5) suggest that humans take correlations between the distractors into account when inferring the presence of a target, but in a suboptimal manner. In particular, people might be assuming that correlations are non-zero even when they are not. Such an assumption of structure in a visual scene could be sensible in light of the prevalence of structured scenes in nature. Similar overestimations of low correlations have been reported in the temporal domain [16–19]. Hence, the suboptimality that we find in our laboratory experiment may reflect an optimal adaptation to the natural world. We note, however, that the assumed correlations seemed to vary between subjects, and were difficult to estimate precisely from the data (See S1B Appendix). Moreover, it does not seem that people overestimate a correlation of zero by enough to account for set size effects in visual short-term memory, as was recently proposed [30][31].

Variable-precision models with a standard encoding stage (VP4) fitted the data reasonably well, except in the *ρ* = 1 condition. To fit all conditions well, we had to construct an ad-hoc model (VP5) in which mean precision depends on correlation condition. In this model, mean precision was estimated higher in the homogeneous (*ρ* = 1) condition; this might be due to a texture detection or other gist mechanism that we do not explicitly model. The difference in mean precision between the homogeneous and the heterogeneous conditions at the surface seems inconsistent with the result shown in Fig 9a of [11], where we did not find a difference. However, this might be due to the fact that in that paper, we assumed the optimal model (VP1) and did not test whether subjects correctly assumed zero correlation.

Of course, the present study is still a far cry from studying the effect of stimulus structure on decision-making in natural scenes, for several reasons. First, the set size used in our experiment was small and known to the observer, while natural visual search tasks often involve a large and unknown number of distractors. Second, our subjects were instructed to maintain fixation, which rarely happens when performing visual search tasks in daily life. Third, natural search targets are often defined by a conjunction of features (e.g., “find the red car-shaped object”). Future work will have to address how well our results generalize to tasks with larger set sizes, free viewing conditions, and conjunction targets. Finally, natural scene statistics are characterized by complex, high-dimensional distributions, making simplified approaches difficult. In particular, the stimuli that we use do not have the complexity of natural stimuli. In a naturalistic model of simple shapes with occlusion, called the dead-leaves model, analytical expressions have been derived for the image values given the world states [33]. It would be interesting to examine to what extent human observers incorporate such statistics in their decision-making.

## Supporting Information

S1 Appendix Optimal decision rule, Model fitting, and model comparison. (DOCX)

S1 Fig Bayesian information criterion results parallelling the Akaike information criterion results in the main text. Higher values mean that the model is worse. (a) Companion to Fig 5. BIC differences between the EP models and their corresponding VP models for each subject (left) and averaged over subjects (right). (b) Companion to Fig 6a. BIC differences between the VP models and the VP4 (most general) model. VP4 outperforms VP1, VP2, and VP3 by 26±13, 103±38, and 47±16 respectively. (c) Companion to Fig 10a. BIC differences between the VP models and the VP5 model for each subject (left) and averaged across subjects (right). The VP5 model outperforms the VP1, VP2, VP3, and VP4 models by 66±25, 142±52, 86±34, and 39±22 respectively.
(TIF)

S2 Fig Model recovery analysis. Results of model comparisons obtained by comparing the fits of the four VP models (rows) to data generated by each model (columns). The color and number in a cell indicate a model’s AIC (a) or BIC (b) value relative to the best fitting model. A value of zero on the diagonal indicates that the model used to generate the data was correctly found to be the most likely model to have generated those data.
(TIF)

S1 Table Parameter estimates. Means and standard error means of the maximum-likelihood estimates of all parameters in all models, as well the tested ranges of the parameters.
(DOCX)

S2 Table Parameter recovery analysis for the VP4 model. Mean, standard error mean, and 95% confidence interval for **ρ**_assumed_ estimates.
(DOCX)
