# Supplementary material for: Do People Take Stimulus Correlations into Account in Visual Search?
Source: PLoS One. 2016 Mar 10;11(3):e0149402. doi: 10.1371/journal.pone.0149402 (PMC4786311; doi:10.1371/journal.pone.0149402)
Supplement: S1 Appendix — (DOCX) [file pone.0149402.s001.docx]

Supporting Information

S1 Appendix.

A. Optimal decision rule

Here, we provide a detailed derivation of the optimal decision rule. Suppose a target is present at the location *j*, for $j\epsilon\left\{ 1,2,\ldots N \right\}$, then

$p\left(s_{j}|T=1\right)=\delta\left(s_{j}\right)$.

We compute the likelihood function, *p*(**x**|*T*) by marginalizing over both **T**=(*T*_1_,…, *T_N_*) and **s**:

.

Note that and, where, vector **1***_j_* has *j*^th^ component as 1 and others as zero, and **0***_N_* denotes a zero vector of length *N*. Following this marginalization, we compute the log-likelihood ratio:

.

Next, we decompose the vector **s** into target stimulus, *s_j_* and distractors, **s**\*_j_*, and similarly break the vector **x** into a target measurement, *x_j_* and distractors measurements, **x**\*_j_*.

We denote by **Σ**_x_ the covariance matrix of the measurements conditioned on the stimuli, which takes the form of an *N*×*N* diagonal matrix with entries *σ*_1_^2^,…, *σ_N_*^2^ on the diagonal and zeros everywhere else. We define **Σ**x\*_j_* as the matrix obtained by removing the *j*^th^ row and *j*^th^ column from **Σ**_x_ so that *p*(*x_j_*|*s_\j_*) = * *(*x_j_*;*s_\j_*, **Σ**_x_*_\j_*). Thus, we obtain

.

We use the fact that products and integrals of multivariate normal distributions are also multivariate normal distributions. In our case, we define **C** = **Σ**_s_+ **Σ**_x_ and **C**\*_j_* = **Σ**s\*_j_*+ **Σ**x\*_j_*. In the case of a positive definite covariance matrix, we integrate and obtain the following expression for the log-likelihood ratio,

.

We further simplify the above equation by computing the inverses of the matrices **C** and **C**\*_j_* using the Woodbury-Sherman formula. Specifically, we obtain

,

where .

The inverse of **C**\*_j_* is obtained similarly by replacing *α* by *α*\*_j_* in the above definition of **C**^-1^. We substitute the above inverse formulae for **C** and **C**\*_j_*, and simplify expressions further. In addition, we assume a uniform prior for *T* to obtain the following expression for the log posterior ratio:

.

B. Model fitting

We fitted the models through maximum-likelihood estimation of their parameters. The likelihoods were based on the raw data (not based on summary statistics such as the ones shown in Figs. 2, 3, and 4). We numerically estimated the likelihoods through Monte Carlo simulations. This means that for a given model, a given parameter combination, and a given subject, we performed the following procedure. For each trial that the subject experienced, we simulated 3000 measurement vectors **x** of the actually presented stimuli on that trial, using the process dictated by the model. We applied the decision rule of the model to each of these measurement vectors to obtain 3000 simulated responses. We used the proportion of these responses that were equal to the subject’s actual response on this trial as an approximation of the model’s probability of the subject’s response. In order to avoid numerical problems, proportions of 0 and 1 were set to 1/3000 and 2999/3000, respectively. The sum of the logarithm of the estimated probability of the subject’s response over all trials in the experiment was an approximation to the log likelihood of the parameter combination and the model. We repeated this log likelihood estimation process for all parameter combinations on a parameter grid with ranges as given in S1 Table. The parameter combination for which the log likelihood was highest was taken as an approximation of the maximum-likelihood estimates of the parameters (see S1 Table). The parameter estimates of the VP4 were used to obtain fits to the subject’s summary statistics in Fig. 7b.

We performed a parameter recovery analysis for the VP4 model to assess the bias in estimating **ρ**_assumed_. We generated 20 synthetic data sets from model VP4 with the number of trials representative of the subject data sets in the experiment. We chose **ρ**_assumed_ **=**(*α*,*β*,*γ*,*δ*)**=ρ**=(0,⅓,⅔,1) and drew other parameters ($\bar{J}$,*τ* and *p*_present_) from a multivariate Gaussian distribution with mean and variance computed from maximum-likelihood estimates of the subjects, and rejected if negative or for p_present_, greater than 1. We fitted these 20 synthetic data sets using the VP4 model. Mean, standard error mean, and 95% confidence interval for **ρ**_assumed_ estimates are given in S2 Table.

We obtained the ML values of **ρ**_assumed_, and did not obtain credible intervals. S2 Table suggests biases in parameter estimation, especially when **ρ**_assumed_ =0. These results suggest that parameter estimates obtained from subject data need to be interpreted carefully; we discuss these concerns in the main text.

C. Model comparison

We used the Akaike [1] and Bayesian [2] information criteria to compare our models. These criteria are based on the maximum value of model-likelihood and penalize a model for additional parameters. Specifically, AIC = −2 log *L** + 2*k*, and BIC = −2 log *L** + *k* log(*n*), where *L** is the log of the maximum likelihood, *k* is the number of model parameters, and *n* is the number of trials. AIC results are reported in the main text. We report the BIC results in Fig. S1.

We also performed a model recovery test to assess the validity of AIC and BIC as measures for distinguishing the different models considered in Table 1. We generated 11 synthetic data sets or fake subjects for each model with 900 trials in each of the four experimental sessions (see Experimental Methods). The number of synthetic data sets and other parameters are chosen to be representative of the subject data sets on the experiment. We generated the synthetic data from a model using maximum likelihood parameter estimates of the subjects ensuring that the statistics of the synthetic data sets are representative of those of subject data. We fitted all 8 models to each synthetic data set. Since VP models better fit the data relative to EP models, we only present the model recovery results for the VP models in Fig. S2.

Based on subject-averaged BIC values, the generative model or correct model was selected in all 4 cases (S2 Fig. b). However, in case of AIC, the correct model was selected in 3 out of 4 cases and with a relatively lower winning difference. The most flexible model VP4 tends to win in all cases as the AIC correction is not large.

**References**

1. Akaike, H. (1974). "A new look at the statistical model identification." IEEE Transactions on Automatic Control **19**(6): 716-723.
2. Schwarz, G. E. (1978). "Estimating the dimension of a model." Annals of Statistics **6**(2): 461-464.
