# Supplementary figures and images for: Do People Take Stimulus Correlations into Account in Visual Search?
Source: PLoS One. 2016 Mar 10;11(3):e0149402. doi: 10.1371/journal.pone.0149402 (PMC4786311; doi:10.1371/journal.pone.0149402)

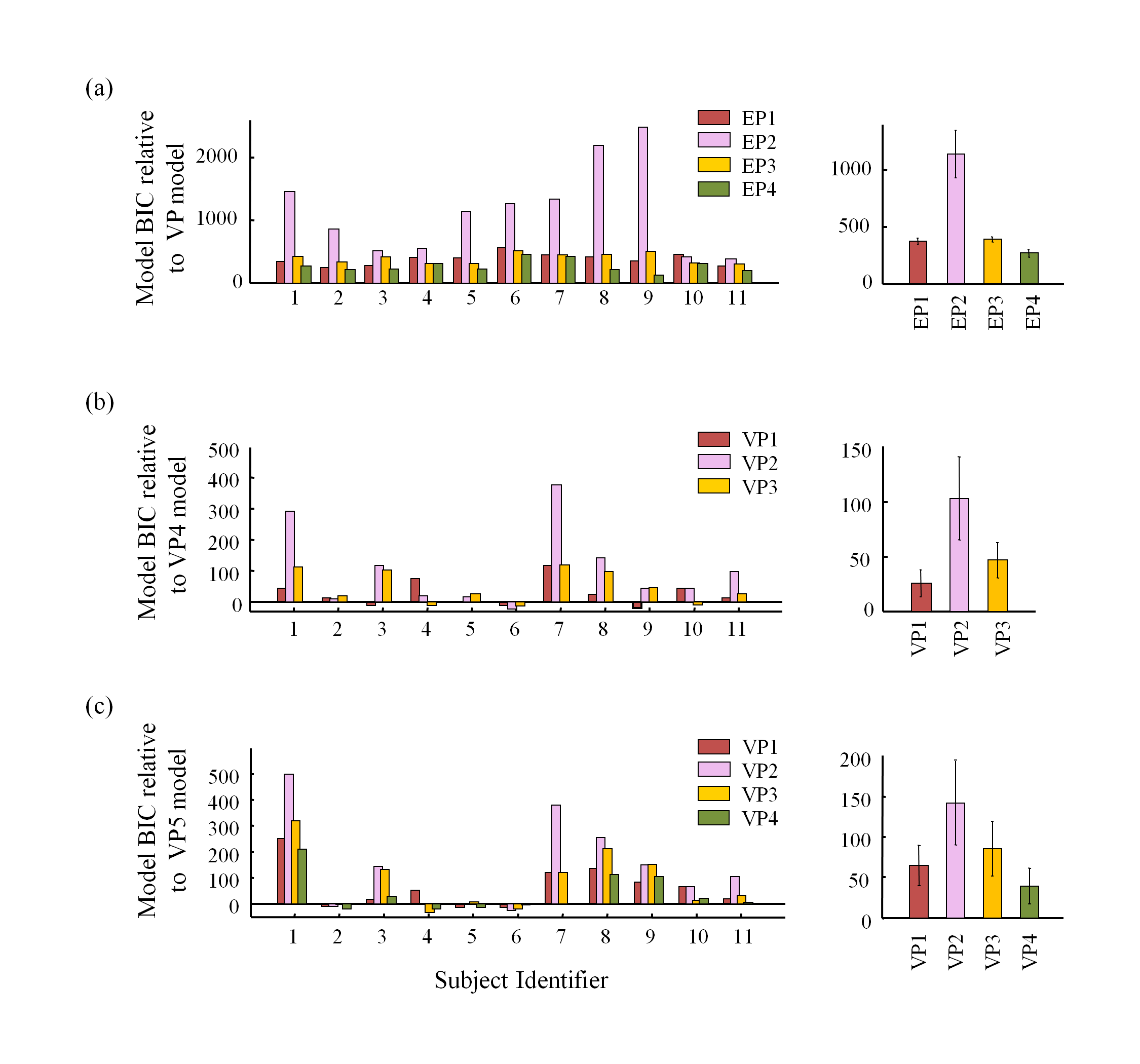

Supplement: S1 Fig — Higher values mean that the model is worse. (a) Companion to Fig 5. BIC differences between the EP models and their corresponding VP models for each subject (left) and averaged over subjects (right). (b) Companion to Fig 6a. BIC differences between the VP models and the VP4 (most general) model. VP4 outperforms VP1, VP2, and VP3 by 26±13, 103±38, and 47±16 respectively. (c) Companion to Fig 10a. BIC differences between the VP models and the VP5 model for each subject (left) and averaged across subjects (right). The VP5 model outperforms the VP1, VP2, VP3, and VP4 models by 66±25, 142±52, 86±34, and 39±22 respectively. (TIF) [file pone.0149402.s002.tif]

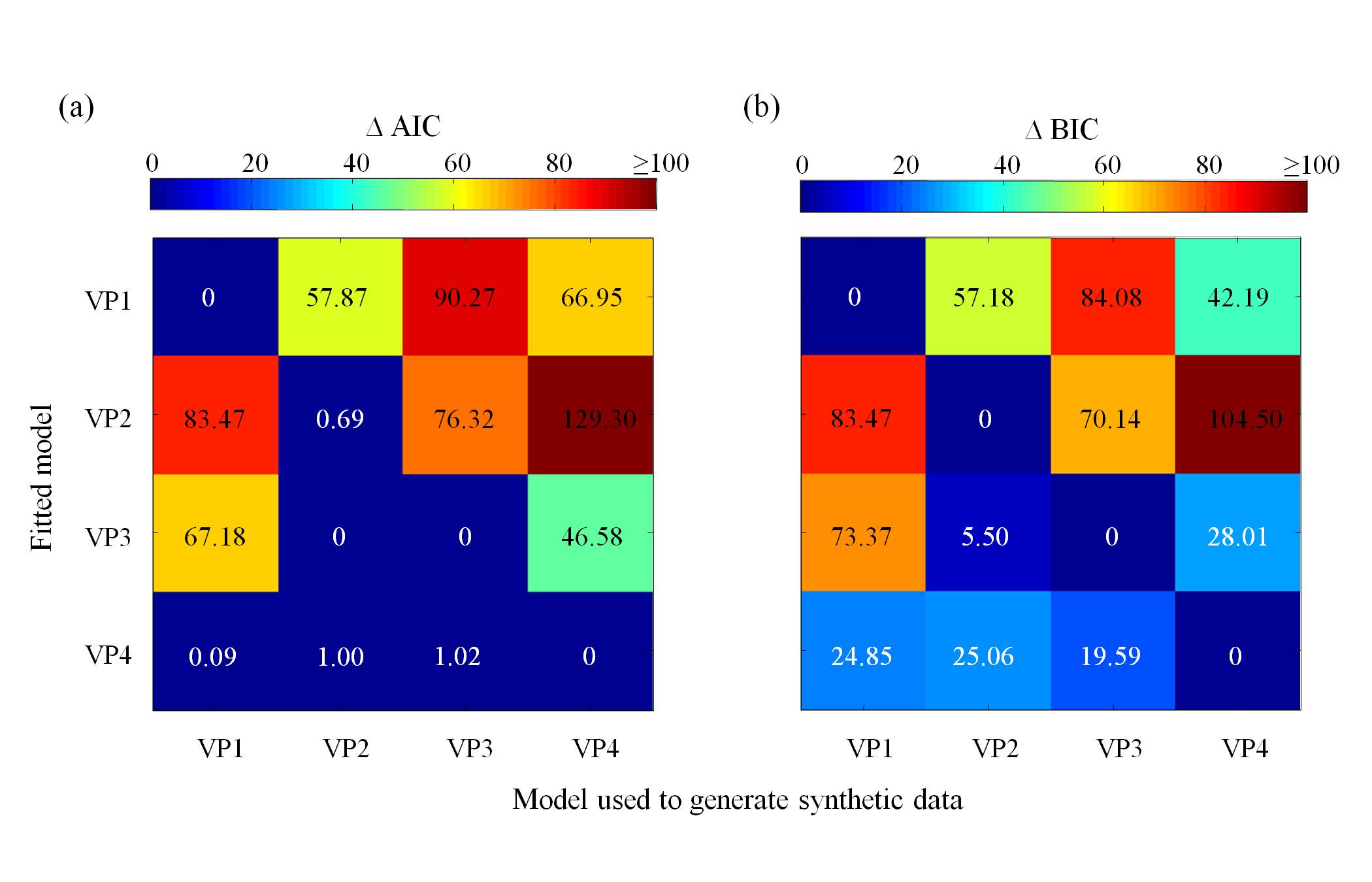

Supplement: S2 Fig — Results of model comparisons obtained by comparing the fits of the four VP models (rows) to data generated by each model (columns). The color and number in a cell indicate a model’s AIC (a) or BIC (b) value relative to the best fitting model. A value of zero on the diagonal indicates that the model used to generate the data was correctly found to be the most likely model to have generated those data. (TIF) [file pone.0149402.s003.tif]
