# Supplementary material for: Do People Take Stimulus Correlations into Account in Visual Search?
Source: PLoS One. 2016 Mar 10;11(3):e0149402. doi: 10.1371/journal.pone.0149402 (PMC4786311; doi:10.1371/journal.pone.0149402)
Supplement: S1 Table — Means and standard error means of the maximum-likelihood estimates of all parameters in all models, as well the tested ranges of the parameters. (DOCX) [file pone.0149402.s004.docx]

**S1 Table. Parameter estimates.** Means and standard error means of the maximum-likelihood estimates of all parameters in all models, as well the tested ranges of the parameters.

| **Model** | **Parameter** | **Mean±SEM** | **(Min, Max)** |
| --- | --- | --- | --- |
| EP1 | *p*_present_  *J* | 0.476±0.004  0.031±0.006 | (0.4,0.6)  (0.02,1.2) |
| EP2 | *p*_present_  *J* | 0.470±0.007  0.096±0.016 | (0.4,0.6)  (0.02,1.2) |
| EP3 | *p*_present_  *J*  α | 0.460±0.004  0.041±0.014  0.642±0.049 | (0.4,0.6)  (0.02,1.2)  (0,1) |
| EP4 | *p*_present_  *J*  *α*  *β*  *γ*  *δ* | 0.466±0.004  0.033±0.010  0.552±0.050  0.527±0.035  0.600±0.039  0.894±0.042 | (0.4,0.6)  (0.02,1.2)  (0,1)  (0,1)  (0,1)  (0,1) |
| VP1 | *p*_present_ $\bar{J}$   *τ* | 0.507±0.003  0.115±0.021  0.734±0.297 | (0.4,0.6)  (0.01,1.5)  (0.1,3.5) |
| VP2 | *p*_present_ $\bar{J}$   *τ* | 0.521±0.006  0.275±0.064  2.330±0.417 | (0.4,0.6)  (0.01,1.5)  (0.1,3.5) |
| VP3 | *p*_present_ $\bar{J}$   *τ*  *α* | 0.510±0.005  0.190±0.036  1.317±0.296  0.539±0.078 | (0.4,0.6)  (0.01,1.5)  (0.1,3.5)  (0,1) |
| VP4 | *p*_present_ $\bar{J}$   *τ*  *α*  *β*  *γ*  *δ* | 0.497±0.005  0.102±0.019  0.569±0.295  0.452±0.080  0.385±0.078  0.509±0.087  0.812±0.089 | (0.4,0.6)  (0.01,1.5)  (0.1,3.5)  (0,1)  (0,1)  (0,1)  (0,1) |
| VP5 | *p*_present_ $\bar{J}_{1}$  | 0.504±0.004  0.098±0.016 | (0.4,0.6)  (0.01,1.5) |
|  | $\bar{J}_{2}$ | 0.094±0.017 | (0.01,1.5) |
|  | $\bar{J}_{3}$ | 0.100±0.018 | (0.01,1.5) |
|  | $\bar{J}_{4}$ | 0.238±0.062 | (0.01,1.5) |
|  | *τ*  *α*  *β*  *γ*  *δ* | 0.569±0.172  0.433±0.074  0.394±0.082  0.482±0.090  0.794±0.086 | (0.1,3.5)  (0,1)  (0,1)  (0,1)  (0,1) |
