# Supplementary material for: Do People Take Stimulus Correlations into Account in Visual Search?
Source: PLoS One. 2016 Mar 10;11(3):e0149402. doi: 10.1371/journal.pone.0149402 (PMC4786311; doi:10.1371/journal.pone.0149402)
Supplement: S2 Table — Mean, standard error mean, and 95% confidence interval for ρassumed estimates. (DOCX) [file pone.0149402.s005.docx]

**S2 Table: Parameter recovery analysis for the VP4 model.** Mean, standard error mean, and 95% confidence interval for **ρ**_assumed_ estimates.

| **Parameter** | **True value** | **Maximum likelihood estimate** | |
| --- | --- | --- | --- |
|  |  | **Mean±SEM** | **95% Confidence Interval** |
| *α* | 0 | 0.14±0.026 | [0.133,0.157] |
| *β* | 0.333 | 0.30±0.035 | [0.284,0.316] |
| *γ* | 0.667 | 0.66±0.015 | [0.645,0.664] |
| *δ* | 1 | 0.98±0.008 | [0.980,0.987] |
